# Effects of monopolar pulsed-capacitive dielectric radiofrequency diathermy in patients with chronic low back pain: a randomised clinical trial

**DOI:** 10.1038/s41598-024-64832-9

**Published:** 2024-06-18

**Authors:** Inmaculada Carmen Lara-Palomo, Ana María Capel-Alcaraz, Héctor García-López, Adelaida María Castro-Sánchez, Manuel Albornoz-Cabello

**Affiliations:** 1 Department of Nursing, Physical Therapy and Medicine, University of Almeria, Ctra. Sacramento s/n La Cañada de San Urbano, 04120 Almería, Spain; 2 Department of Physiotherapy, University of Seville, 41004 Seville, Spain

**Keywords:** Chronic low back pain, Diathermy, Electrophysical agent, Radiofrequency, Simulated therapy, Rehabilitation, Placebo effect

## Abstract

Monopolar capacitive diathermy is a physiotherapy technique that uses high-frequency currents to generate heat in deep tissues. This heat can have several therapeutic effects, especially in the treatment of chronic low back pain (CLBP), however, until now there is little evidence of this type of diathermy. The purpose was to evaluate the efficacy of a pulsed monopolar dielectric radiofrequency diathermy (PRF)-capacitive type versus simulated treatment on symptomatology of patients with CLBP. A single-blind randomised controlled trial was conducted. Sixty patients with CLBP were randomly assigned to a PRF-capacitive or a simulated treatment group. All participants received 3 sessions per week for 3 weeks. Disability, pain intensity, movement phobia, lumbar anteflexion, quality of life, and sleep quality were assessed at baseline, after treatment, and at two months. The application of 9 sessions of PRF-capacitive showed significant improvements compared to simulated therapy during the entire follow-up for disability (F = 26.99, *p* < 0.001), pain intensity (F = 0.550, *p* < 0.001), the quality of life components of physical function (F = 0.780, *p* < 0.001), social function (F = 0.780, *p* < 0.001) and mental health (F = 0.858, *p* = 0.003) and for sleep duration (F = 0.863, *p* = 0.004).

## Introduction

Chronic low back pain (CLBP) is a common global problem. The point prevalence of CLBP in 2017 was estimated to be about 7.5% of the global population, or around 577.0 million people^1^. Between 85 and 95% of people presenting to primary care providers do not have a specific identifiable pathoanatomical origin for their pain^2^. Nonspecific CLBP is defined as pain or discomfort in the area below the lower 12th rib and the inferior gluteal fold, lasting for at least 12 weeks, with no identifiable specific spinal disease, radiculopathy or nerve root pain^3^. CLBP is therefore a complex, common and distressing problem that has a major impact on the world’s population, particularly in low and middle-income countries, varying widely among individuals according to an array of biophysical, psychological and social factors^4,5^.

The Global Burden of Disease 2016 study^4^ reaffirmed that LBP is one of the five main causes of years lived with disability internationally, which is worsening due to ageing and growing populations^6^. Furthermore, with the increasing rates of obesity, the fast growth of industrialisation, and the consequent reduction in physical activity, this problem is increasingly affecting urban areas^5,7^. It is estimated that at least half of the general population will experience low back pain at some point in their lives, with 5–10% being chronic^8^.

According to some theories, one of the mechanisms for the pathophysiology of CLBP is that after chronic musculoskeletal injury, inflammatory mediators are released that promote the sensitisation of nociceptors, often resulting in symptoms of hyperalgesia to mechanical stimuli, as well as alterations in physical and psychological functioning and quality of life^9−13^. Currently, there are studies that demonstrate that patients with persistent chronic pain without tissue damage have alterations in brain regions involved in cognitive, sensory, and emotional modulation of pain^14–17^.

Among electrophysical agents, radio waves, ultrasound, and tecar therapy have been widely used in the treatment of CLBP^18–22^, however, this is little evidence of other types of diathermy. Radiofrequency diathermy of the pulsed monopolar dielectric (PRF)-capacitive type is a form of high-frequency electromagnetic stimulation used in clinical practice to reduce pain and inflammation and improve tissue healing^20–24^. Energy absorption between 450 and 1000 kHz results primarily in the heating of tissue, which results in energy transfer from PRF to deeper tissues, and subsequent biological effects^23–26^. Previous studies have shown that the use of PRF can have beneficial effects in the relief of chronic pain, including patellofemoral pain^27^, knee osteoarthritis^28^, tendinopathies^29^ and fibromyalgia^30^, by interfering with the transmission of pain signals and reducing local inflammation^31,32^. However, little is known about the effects of this type of diathermy in patients with CLBP.

According to the theoretical scientific basis and clinical evidence, to evaluate the effects of a capacitive treatment with PRF (Pulsed Radiofrequency) in patients with non-specific CLBP, we propose the following scientific hypothesis: “Capacitive PRF treatment is more effective in reducing pain and improving functionality in patients with non-specific CLBP compared to a sham control group.”

On the other hand, it was compared with a simulated control group because we believe that it is essential to establish the efficacy of capacitive treatment with PRF, eliminating the placebo effect and providing a solid basis for comparing the clinical results obtained.

## Methods

### Design

This study was a single-blind randomized controlled clinical trial (allocation ratio 1:1) with intention-to-treat analysis, designed and conducted according to CONSORT guidelines (Appendix 1). The protocol was approved by the local human research bioethics committee of the University of Almeria (Spain) on 2021 (UALBIO2021/005) and complied with the 2013 modification of the It was performed following the Helsinki Declaration and was registered in Clinicaltrials.gov on 22/07/2022 (Registration number: NCT05471258). All subjects signed an informed consent form.

### Participants

A total of 68 patients aged between 18 and 65 years, diagnosed with CLBP of more than 3 months duration and with a pain intensity of at least 3 points, were studied between March 2022 and October 2022 through an advertisement displayed on social networks. The study was conducted at the Clinical Physiotherapy Unit of the Faculty of Health Sciences of the University of Almeria. Patients were assessed and enrolled in this study if they met the following selection criteria.

Inclusion criteria

- Men and women between the ages of 18 and 65.
- Diagnosed by a specialist CLBP physician.
- 3 or more in pain intensity by Visual Analog Scale (VAS).
- Acceptance and signature of informed consent for voluntary participation in the research study.
- Not undergoing any type of physical therapy or pharmacological treatment.
- Acceptance to attend 90% of the treatment sessions.

Exclusion criteria

- Undergoing rehabilitation or pharmacological treatment for the pathology of lumbar origin.
- Participants could not have previously received diathermy treatment, in any of its types (capacitive or resistive).
- Alterations in sensitivity or coagulation.
- Thermal sensitivity problems.
- Presence of osteosynthesis material at lumbar level.
- Cardiac, epilepsy or tumour complications.
- Patients who have recently undergone radiotherapy.
- Non-acceptance of informed consent or non-attendance at more than 90% of the treatment sessions.

### Outcome measures

**Figure 1:**
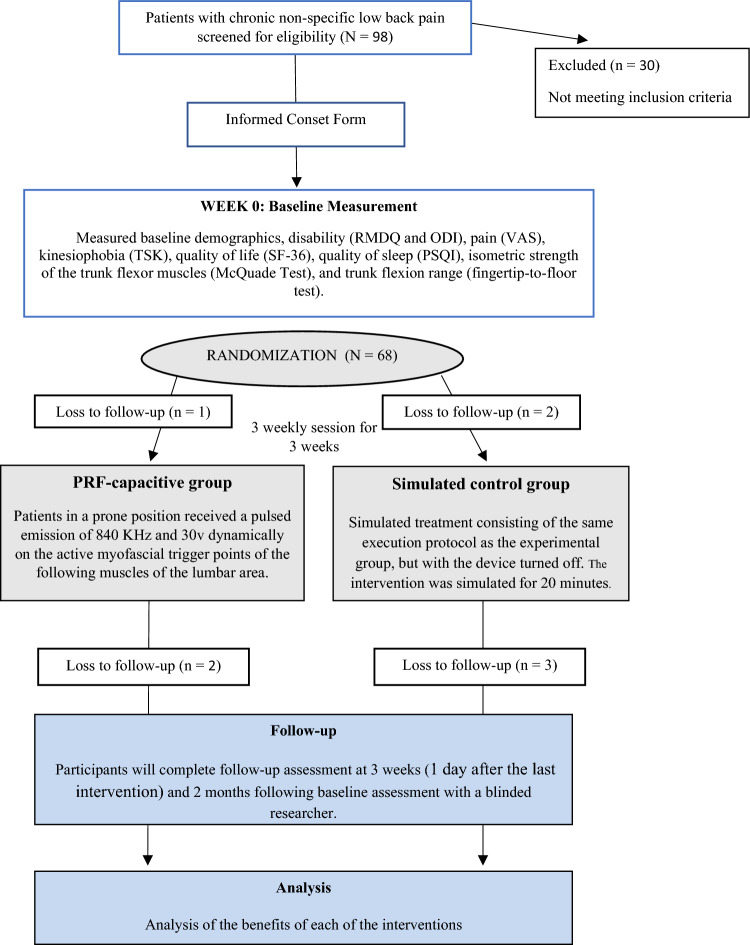
Design and flow of participants through the trial. ODI, Oswestry Disability Index; MDR, Monopolar Dielectric Diathermy by emission of Radiofrequency; PSQI, Pittsburgh Sleep Quality Index; RMQ, Roland–Morris Low Back and Disability Questionnaire; TSK, Tampa Scale for Kinesiophobia; VAS, Visual Analogue Scale.

At the start of the study, all participants provided clinical and demographic data such as age, sex, weight, height, education and clinical presentation of symptoms, and completed a series of self-report measures. Physical examination was performed by a blinded assesor who was unaware of the participants’ treatment assignment. Outcome measures were assessed before randomisation (baseline assessment), immediately after treatment (1 day after the last intervention) and at two months after the end of the intervention (short-term follow-up) (Fig. 1).

A 30% improvement was considered a useful threshold to identify a clinically meaningful improvement in each of the measures^33^.

#### Primary outcome measures

- Roland Morris Disability Questionnaire (RMDQ): This questionnaire consists of 24 items that rate limitations in different activities of daily living attributed to low back pain, such as walking, bending, sitting, lying down, dressing, sleeping, self-care and activities of daily life. Disability is rated from 0 points (best) to 24 points (worst)^34^.

- Visual Analogue Scale (VAS): assesses pain intensity with a score from 1 to 10, where the highest pain is 10^35^.

#### Secondary outcome measures

- *Oswestry Low Back Pain Disability Index (ODI)* a low back pain specific questionnaire, which measures limitations in the patient’s day-to-day life. The total score is a percentage from 0 to 100%, rating the patient as minimal functional limitation (0–20%); moderate functional limitation (20–40%); severe functional limitation (40–60%); disability (60–80%); maximum functional limitation (more than 80%)^34^.
- *Tampa Kinesiophobia Scale (TSK)* 17-item questionnaire measuring the fear of movement and injury relapse. Each item is scored on a four-point Likert scale ranging from “strongly disagree”^1^ to “strongly agree”^4^. The total score ranges from 17 to 68 points^36^.
- *SF-36 quality of life questionnaire* A health survey consisting of 36 questions indicating self-perceived health-related quality of life. It consists of eight domains (physical function, physical role, bodily pain, general health, vitality, social function, emotional role, and mental health) and two summary scores: physical health and mental health. Scores range from 0 to 100%^37^.
- *Pittsburgh sleep quality index (PSQI)* 10-item questionnaire with a total of 19 questions related to sleep habits in the previous month. The questions are divided into 7 areas, each with a score between 0 and 3 points. The overall score ranges from 0 (no difficulty sleeping) to 21 points (severe difficulty sleeping)^38^.
- *McQuade test* This test assesses the isometric strength of the trunk flexor muscles. The patient is placed in the supine position and asked to flex the head and shoulders until the scapula is lifted off the table. The number of seconds they hold this position is recorded, the maximum being 120 s^39^.
- *Anterior trunk flexion* Measures lumbar mobility in trunk flexion from a standing position. Standing up with the legs streched out, the subject is asked to lean forward and try to touch the ground, stopping when pain or limitation of movement appears. The distance between the toes and the ground is measured in centimetres^40^.

### Sample size

The sample size was based on estimates established by Willian^41^. The calculations were based on the detection of 2.5 point differences in RMDQ score (minimum clinically important difference), assuming a standard deviation of 2.5 points, a 2-tailed test, an alpha (α) of 0.05 and an objective power (beta) of 85%. The estimated sample size was 60 subjects (30 per group).

### Randomisation

After the initial assessment, participants who met the eligibility criteria were randomised in a 1:1 ratio to receive either (i) high-frequency therapy using PRF-training signals on myofascial trigger point symptomatology or (ii) simulated control treatment. Both groups were treated by two physiotherapists with more than 10 years of experience in managing people with CLBP. Hidden allocation was performed using a computer-generated random table of numbers created prior to the start of data collection by a researcher who was not involved in patient recruitment or treatment. Sequentially numbered individual tokens were prepared with the random allocation. The cards were folded and placed in sealed opaque envelopes. Another therapist, blinded to the initial examination, opened the envelope and proceeded with treatment according to group assignment. The outcome assessor and study statistician were blinded throughout. The computer-generated outcome measures transmitted to the statistician contained no information identifying the group assigned to each patient.

### Interventions

After the randomisation, consenting participants were placed randomly into two groups to receive PRF-capacitive or simulated treatment. All participants received 3 sessions per week for 3 weeks, for a total of 9 sessions. Patients in both treatment groups had to undergo at least 90% of their scheduled treatment sessions to be considered and remain in the intention-to-treat analysis. The nature of the intervention did not allow for the blinding of the physiotherapist and participants. No kinesiotherapy was administered in any group and no self-administered therapeutic exercise was administered. Potential adverse effects were to be reported to the principal investigator. Details of the interventions are provided below.

#### PRF-capacitive group

The experimental group underwent a PRF-capacitive application using the Physicalm® device (Biotronic Advance Develops SL, Granada, Spain). Patients in a prone position received a pulsed emission of 840 kHz and 30v dynamically (rotational and translational movements) on the active myofascial trigger points of the following muscles of the lumbar area: lumbar quadratus lumborum, multifidus and iliocostalis. The time per session was 20 min, 3 sessions were carried out per week for 3 weeks, on Mondays, Wednesdays and Fridays, in total 9 treatment sessions.

#### Simulated control group

The control group was administered a simulated treatment consisting of the same execution protocol as the experimental group, but with the device turned off. The intervention was simulated for 20 min. Three sessions were carried out per week for three weeks, on Mondays, Wednesdays and Fridays, for a total of nine treatment sessions. Once the simulated intervention period was over and follow-up data was collected, a washout period was established and the same treatment was given as for the experimental group.

### Patient and public involvement statement

Patients were not involved in the design, or conduct, or reporting, or dissemination plans of our research.

### Data analysis

The IBM SPSS© version 28.0 software package was used for statistical analysis. A *p*-value < 0.05 was considered statistically significant. After a descriptive analysis, the normal distribution of the variables was tested using the Kolgomorov–Smirnov test. Baseline demographic and clinical variables were compared between the two groups using χ2 tests for categorical data and Student’s t-tests for continuous data. Repeated measures analysis of variance (2 × 3 mixed model ANOVA) was used to analyse time effects between both groups (PRF-capacitive versus simulated) and group interaction effects for all outcome measures (RMDQ, and VAS as primary outcome), between immediately after treatment and two months later. Changes in variable scores within and between groups were measured by 95% confidence interval t-tests for paired or independent samples, as appropriate.

## Results

### Baseline characteristic

For this randomized trial, a total two hundred and fifty (n = 250) patients with LBP were screened for eligibility. Reasons for exclusion are shown in Fig. 1—a patient recruitment and inclusion flow chart. Sixty patients (mean age ± SD: 48.5 ± 9.2; 53.3% male) met the inclusion criteria and were randomized to receive treatment with either PRF-capacitive (N = 30), or simulated therapy (N = 30). There were no significant differences between the two groups at baseline in terms of demographics shown in Table 1. All patients who participated did not receive any other treatments during the process of the study. The Kolmogorov–Smirnov test showed the normal distribution of the numerical variables.

**Table 1:** Patient characteristics at baseline.

Variable	PRF-capacitive group N = 30	Simulated Group N = 30	*P-values*
Gender (m/f)	17/13	15/15	0.31
Age (years)	48.20 ± 10.22	48.77 ± 8.14	0.41
Weight (kg)	77.43 ± 17.05	76.2 ± 10.15	0.37
Height (cm)	169.07 ± 0.09	168 ± 0.09	0.32
Education level (N)			0.41
No studies	0	0	–
School level	1	0	–
Bachelor level	11	12	–
University level	18	18	–
Intake of medication			0.14
None	15	13	–
Once a week	6	7	–
Once every two days	7	2	–
Once or twice a day	2	7	–
Three or more times a day	0	1	–

*Values are expressed as absolute and relative frequencies (n = 60) for categorical variables and as means ± standard deviations for continuous variables. No differences between groups (*P* > 0.050).

### Self-reported outcomes

#### Disability

Group * Time interaction for the 2 × 3 mixed ANOVA test showed significant differences only for RMDQ (F = 26.99, *p* < 0.001). However, analysis between the two groups revealed statistically significant differences at the 3-week post-treatment evaluation and the two-month follow up, regarding the main outcome measure, in the RMDQ (Post- treatment: F = 2.212, *p* < 0.001; 2-month follow-up: F = 1.778, *p* < 0.001), and for the ODI (Post-treatment: F = 0.678, *p* < 0.001; follow up: F = 0.006, *p* < 0.001) (Table 2).

**Table 2:** Baseline, immediate post-treatment, two months follow-up, and change score between groups for disability, pain, kinesiophobia, isometric resistance of abdominal muscles and spinal mobility in flexion.

Outcome/group	PRF-capacitive group (n = 30)	Simulated group (n = 30)	Mean Differences and CI between groups over time	*p* Value
Self-reported measures				
RMD (0–24)				
Baseline	8.03 ± 3.53	9.20 ± 3.63	− 1.17 (− 3.02, 0.68)	
Post-Treatment (3 Week)	2.67 ± 2.56	7.60 ± 3.50	− 4.93 (− 6.52, − 3.34)	< 0.001*
Follow-up (2 Months)	3.23 ± 2.31	5.93 ± 2.84	− 2.70 (− 4.04, − 1.36)	< 0.001*
ODI (0–50)				
Baseline	27.20 ± 10.67	26.57 ± 9.36	0.63 (− 4.55, 5.82)	
Post-Treatment (3 Week)	9.93 ± 6.88	23.07 ± 8.87	− 13.13 (− 17.24, − 9.03)	< 0.001*
Follow-up (2 Months)	10.87 ± 7.99	23.27 ± 8.32	− 12.40 (− 16.62, − 8.18)	< 0.001*
VAS (0–10 points)				
Baseline	6.42 ± 2.06	6.38 ± 1.69	0.03 (− 0.94, 1.00)	
Post-Treatment (3 Week)	2.75 ± 2.02	4.50 ± 1.71	− 1.75 (− 2.72, − 0.78)	< 0.001*
Follow-up (2 Months)	3.70 ± 1.81	3.28 ± 1.47	0.42 (− 0.44, 1.27)	0.166
TSK (17–68)				
Baseline	29.67 ± 6.68	29.13 ± 5.62	0.53 (− 2.66, 3.72)	
Post-treatment (3 week)	22.20 ± 6.09	27.80 ± 4.85	− 5.60 (− 8.45, − 2.75)	< 0.001*
Follow-up (2 Months)	24.50 ± 7.01	31.90 ± 5.86	− 7.40 (− 10.74, − 4.06)	< 0.001*
Physical outcomes				
McQuade Test (s)				
Baseline	48.60 ± 24.94	46.40 ± 21.25	2.20 (− 9.76, 14.18)	
Post-Treatment (3 Week)	63.68 ± 25.41	50.47 ± 18.14	13.22 (1.81, 24.63)	0.012*
Follow-up (2 Months)	66.23 ± 27.84	53.00 ± 21.36	13.23 (0.41, 26.06)	0.022*
Finger-to-floor distance (cm)				
Baseline	14.23 ± 8.44	13.53 ± 5.94	0.70 (− 3.07, 4.47)	
Post-Treatment (3 Week)	8.30 ± 6.81	13.80 ± 6.01	− 5.50 (− 8.82, − 2.18)	< 0.001*
Follow-up (2 Months)	8.37 ± 6.52	12.80 ± 6.51	− 4.43 (− 7.80, − 1.07)	0.005*

Values are expressed as mean ± standard deviation for baseline, immediate post-treatment and two months follow up and as mean (95% confidence interval) for between-group change scores.*p ˂ 0.05 significant ANOVA adjusted from baseline values for differences among group.

*MDR, Monopolar Dielectric Radiofrequency; RMD, Roland–Morris Low Back and Disability Questionnaire; ODI, Oswestry Disability Index; VAS, Visual Analogue Scale; TSK, Tampa Scale for Kinesiophobia.

Significant values are in bold.

When analysing the two groups, the PRF-capacitive group showed greater changes from baseline between the third week and the second month: RMDQ (post: 5.37, *p* < 0.001; follow-up: − 0.57, *p* < 0.030) and ODI (post: 17.27, *p* < 0.001; follow-up: − 0.93, *p* < 0.37), by contrast, the simulated group changes were not as significant at two months for ODI (follow up: − 0.2, *p* = 0.327).

#### Pain intensity

The 2 × 3 mixed ANOVA did not reveal a significant difference between the VAS scores of both groups (F = 0.550, *p* < 0.001). At 3 weeks post-treatment, the groups revealed differences in pain intensity (F = 0.617, *P* < 0.001), where the PRF-capacitive group displayed a greater decrease. No significant differences between the groups were found at the two-month follow-up (Table 2).

During the analysis of the groups, the score change in pain intensity was significantly greater in the PRF-capacitive group than in the simulated group at 3 weeks post-treatment: the PRF-capacitive group (post: 3.67, *p* < 0.001), the simulated group (post: 1.88, *p* < 0.001). Nonetheless, the decrease in pain was similar in both groups at the 2-month follow-up.

#### Kinesiophobia

The ANOVA analysis did not indicate statistically significant differences between the groups for kinesiophobia. However, analysis did show statistically significant differences between the groups at all follow-up periods (post: F = 0.891, *p* < 0.001; follow-up: F = 0.693, *p* < 0.001).

An analysis of repeated-measures showed that the PRF-capacitive group gradually experienced a greater decrease during the week 3 post-treatment evaluation (PRF-capacitativa: 7.47, *p* < 0.001; Simulated: 1.33, *p* = 0.33). At the two-month follow-up, the simulated group deteriorated from baseline measures (baseline: 29.13; follow-up: 31.9).

#### Quality of life

Regarding the quality of life, the 2 × 3 mixed-model ANOVA with repeated measurements analysis showed significant time * groups interaction for physical function (F = 0.780, *p* < 0.001), social function (F = 0.780, *p* < 0.001), and mental health subscales (F = 0.858, *p* = 0.003). During the group analysis, significant differences for the following subscales of quality of life on the SF-36 appeared between week 3 of post-treatment evaluation [body pain (F = 3.100, *P* = 0.023), and emotional role (F = 53.003, *p* = 0.002)], and at the two month’s evaluation [social function (F = 29.234, *p* < 0.001), and emotional role (F = 37.563, *p* = 0.004)]. Table 3 shows the differences between the groups for these results.

**Table 3:** Changes baseline, post treatment, and two months follow-up of quality of life SF-36.

Outcome/ Group	PRF-capacitive group (n = 30)	Simulated Group (n = 30)	Mean Differences and CI between groups over time	*p* Value
Quality of life SF-36 questionnaire				
Physical function				
Baseline	77.00 ± 15.95	74.50 ± 13.60	2.50 (− 5.16, 10.16)	
Post-treatment (3 Week)	84.50 ± 13.86	79.00 ± 11.48	5.50 (− 1.08, 12.08)	0.050
Follow-up (2 Months)	81.50 ± 16.09	83.33 ± 12.62	− 1.83 (− 9.31, 5.64)	0.313
Physical role				
Baseline	90.72 ± 14.14	88.33 ± 17.04	2.39 (− 5.70, 10.48)	
Post-treatment (3 Week)	93.06 ± 11.80	94.17 ± 12.60	− 1.11 (− 7.42, 5.20)	0.363
Follow-up (2 Months)	89.06 ± 14.43	94.17 ± 12.60	− 5.11 (− 12.11, 1.89)	0.075
Body pain				
Baseline	58.67 ± 21.09	53.25 ± 18.03	5.42 (− 4.72, 15.56)	
Post-treatment (3 Week)	76.08 ± 17.62	68.08 ± 12.12	8.00 (0.18, 15.82)	0.023*
Follow-up (2 Months)	75.83 ± 17.20	70.25 ± 13.57	5.58 (− 2.42, 13.59)	0.084
General health				
Baseline	64.17 ± 15.20	59.50 ± 14.93	4.67 (− 3.12, 12.46)	
Post-treatment (3 week)	74.17 ± 14.86	69.50 ± 16.37	4.67 (− 3.41, 12.75)	0.126
Follow-up (2 months)	77.33 ± 15.35	72.67 ± 17.06	4.67 (− 3.72, 13.05)	0.135
Vitality				
Baseline	59.17 ± 17.47	57.50 ± 13.69	1.67 (− 6.45, 9.78)	
Post-treatment (3 week)	68.83 ± 14.60	64.83 ± 11.26	4.00 (− 2.74, 10.74)	0.120
Follow-up (2 months)	73.33 ± 17.29	68.67 ± 14.38	4.67 (− 3.55, 12.88)	0.130
Social function				
Baseline	75.00 ± 21.53	68.75 ± 18.20	6.25 (− 4.05, 16.55)	
Post-treatment (3 Week)	81.25 ± 14.95	86.25 ± 13.67	− 5.00 (− 12.41, 2.41)	0.091
Follow-up (2 months)	78.33 ± 17.35	95.83 ± 5.99	− 17.50 (− 24.21, − 10.79)	< 0.001*
Mental health				
Baseline	68.93 ± 13.06	73.47 ± 10.21	− 4.53 (− 10.59, 1.53)	
Post-treatment (3 Week)	80.40 ± 9.42	79.67 ± 11.20	0.73 (− 4.62, 6.08)	0.392
Follow-up (2 Months)	81.73 ± 10.59	85.33 ± 11.12	− 3.60 (− 9.21, 2.01)	0.120
Emotional role				
Baseline	85.67 ± 25.55	77.78 ± 26.75	7.89 (− 5.63, 21.41)	
Post-treatment (Week 3)	97.78 ± 8.45	87.79 ± 16.32	9.99 (3.27, 16.71)	0.002*
Follow-up (2 months)	97.78 ± 8.45	85.56 ± 22.63	12.22 (3.39, 21.05)	0.004*

Values are expressed as mean ± standard deviation for immediate post-treatment, and two months follow up, and as mean (95% confidence interval) for between-group change scores. *p ˂ 0.05 significant ANOVA adjusted from baseline values for differences among groups.

Significant values are in bold.

Regarding comparisons within the groups, both showed significant differences between baseline and post-treatment for all subscales of the SF-36 quality of life questionnaire. However, at two months of the follow-up, the PRF-capacitive group obtained statistically significant differences for subscales of the SF-36 quality of life in terms of physical function (3.00, *p* = 0.036), physical role (4.00, *p* = 0.035), general health (− 3.17, *p* = 0.007), vitality (− 4.50, *p* < 0.001), and mental health (− 1.33, *p* = 0.019). The simulated group obtained statistically significant differences for the subscales in terms of physical function (− 4.33, *p* < 0.001), general health (− 3.17, *p* = 0.039), vitality (− 3.83, *p* = 0.002), social function (− 9.58, p < 0.001), and mental health (− 5.67, *p* < 0.001).

#### Quality of sleep

Regarding quality of sleep, the 2 × 3 mixed-model ANOVA with an analysis of repeated measurements showed significant time * groups interaction for sleep duration (F = 0.863, *P* = 0.004). Analysis of the groups also demonstrated significant differences during the week 3 post-treatment evaluation [daily sleep dysfunction (F = 1.508, *p* = 0.011); sleep disruption (F = 6.593, *p* = 0.034); habitual sleep efficacy (F = 0.000, *p* = 0.003); sleep duration (F = 0.014, *p* < 0.001), and total score PSQI (F = 0.027, *p* < 0.001)]. However, evaluation of the two months for the following subscales of the PSQI quality of sleep showed otherwise [daily sleep dysfunction (F = 25.375, *p* < 0.001); sleep disruption (F = 0.901, *p* = 0.023); habitual sleep efficacy (F = 0.014, *p* < 0.001); sleep duration (F = 0.291, *p* < 0.001), and total score PSQI (F = 0.020, *p* < 0.001)]. Table 4 reflects the differences in outcome between the groups.

**Table 4:** Changes baseline, post-treatment, and two months follow-up of quality of sleep.

Outcome/group	PRF-capacitive group (n = 30)	Simulated Group (n = 30)	Mean differences and CI between groups over time	*p* Value
Pittsburgh sleep quality index (0–21)				
Total score				
Baseline	9.70 ± 4.06	9.43 ± 4.21	0.27 (− 1.87, 2.40)	
Post-Treatment (3 Week)	5.70 ± 3.02	8.60 ± 3.09	− 2.90 (− 4.48, − 1.32)	< 0.001*
Follow-up (2 Months)	4.73 ± 2.64	8.03 ± 2.94	− 3.30 (− 4.74, − 1.86)	< 0.001*
Daily sleep dysfunction				
Baseline	0.87 ± 0.94	0.97 ± 0.85	− 0.10 (− 0.56, 0.36)	
Post-Treatment (3 Week)	0.33 ± 0.55	0.70 ± 0.65	− 0.37 (− 0.68, − 0.06)	0.011*
Follow-up (2 Months)	0.17 ± 0.38	0.67 ± 0.76	− 0.50 (− 0.81, − 0.19)	0.001*
Use of hypnotics for sleep				
Baseline	0.73 ± 1.14	0.77 ± 0.94	− 0.03 (− 0.57, 0.51)	
Post-Treatment (3 Week)	0.63 ± 0.96	0.57 ± 0.77	0.07 (− 0.39, 0.52)	0.384
Follow-up (2 Months)	0.63 ± 1.00	0.37 ± 0.56	0.27 (− 0.15, 0.68)	0.103
Sleep perturbation				
Baseline	1.27 ± 0.58	1.13 ± 0.57	0.13 (− 0.17, 0.43)	
Post-Treatment (3 Week)	1.20 ± 0.55	0.97 ± 0.41	0.23 (− 0.02, 0.49)	0.034*
Follow-up (2 Months)	1.17 ± 0.53	0.90 ± 0.48	0.27 (0.01, 0.53)	0.023*
Habitual sleep efficacy				
Baseline	2.00 ± 0.79	1.63 ± 1.00	0.37 (− 0.10, 0.83)	
Post-Treatment (3 Week)	0.80 ± 1.00	1.50 ± 0.94	− 0.70 (− 1.20, − 0.20)	0.003*
Follow-up (2 Months)	0.77 ± 1.01	1.70 ± 0.95	− 0.93 (− 1.44, − 0.43)	< 0.001*
Sleep duration				
Baseline	1.77 ± 1.04	2.07 ± 0.94	− 0.30 (− 0.81, 0.21)	
Post-Treatment (3 Week)	0.77 ± 0.77	2.30 ± 0.75	− 1.53 (− 1.93, − 1.14)	< 0.001*
Follow-up (2 Months)	0.50 ± 0.63	2.43 ± 0.68	− 1.93 (− 2.27, − 1.59)	< 0.001*
Sleep latency				
Baseline	1.60 ± 1.04	1.63 ± 1.00	− 0.03 (− 0.56, 0.49)	
Post-Treatment (3 Week)	1.13 ± 0.90	1.53 ± 0.97	− 0.40 (− 0.88, 0.08)	0.052
Follow-up (2 Months)	0.93 ± 0.83	1.27 ± 0.87	− 0.33 (− 0.77, 0.11)	0.067
Subjective sleep quality				
Baseline	1.47 ± 0.78	1.23 ± 0.63	0.23 (− 0.13, 0.60)	
Post-treatment (3 week)	0.83 ± 0.65	1.03 ± 0.56	− 0.20 (− 0.51, 0.11)	0.102
Follow-up (2 months)	0.57 ± 0.63	0.70 ± 0.60	− 0.13 (− 0.45, 0.18)	0.201

Values are expressed as mean ± standard deviation for immediate post-treatment, and two months follow up, and as mean (95% confidence interval) for between-group change scores. *p ˂ 0.05 significant ANOVA adjusted from baseline values for differences among groups.

Significant values are in bold.

When comparing the groups, the PRF-capacitive group showed significant differences between baseline and 3 weeks post-treatment for the subscales of the PSQI daily sleep dysfunction (0.53, *p* < 0.001), habitual sleep efficacy (1.20, *p* < 0.001), sleep duration (1.00, *p* < 0.001), sleep latency (0.47, *p* < 0.001), subjective sleep quality (0.63, *P* < 0.001), and total PSQI score (4.00, *p* < 0.001), and two-month follow-up daily sleep dysfunction (0.17, *p* = 0.048), sleep duration (0.27, *p* = 0.015), sleep latency (0.20, *p* = 0.016), subjective sleep quality (0.27, *p* = 0.004), and PSQI total score (0.97, *p* < 0.001). On the other hand, the simulated group experienced significant inner-group changes at 3 weeks from baseline for the PSQI subscales daily sleep dysfunction (0.27, *p* = 0.001), sleep disruption (0.17, *p* = 0.011) sleep duration (− 0.23, *p* = 0.035), subjective sleep quality (0.20, *p* = 0.006), and PSQI total score (0.83, *p* = 0.039), and two-month follow-up use of hypnotics for sleep (0.20, *p* = 0.042), sleep latency (0.27, *p* = 0.009) ,subjective sleep quality (0.33, *p* < 0.001), and PSQI total score (0.57, *p* = 0.049).

### Physical outcomes

Similarly, no statistically significant ANOVA results were achieved for the McQuade test and fingertip-to-floor distance. However, analysis of the groups showed statistically significant differences at 3 weeks post-treatment and the 2 months follow-up: in the McQuade test (post: F = 3.105, *p* = 0.012; follow-up: F = 1.014, p = 0.022) and in the fingertip-to-floor distance test (post: F = 1.597, *p* < 0.001; follow-up: F = 0.240, *p* = 0.005). During post-treatment, the McQuade test scores increased 15.08 s for patients who received PRF-capacitative treatment and 4.06 s for those receiving simulated therapy. The changes were similar in both groups at the follow-up from the baseline.

After treatment, the fingertip-to-floor distance decreased 5.93 cm in the group that received PRF-capacitive treatment and − 0.26 cm in the simulated group. The results were maintained at the follow-up in both groups (see Table 2).

## Discussion

We set out to conduct a unique study to evaluate the efficiency of PRF-capacitive treatment with 840 kHz pulsed emission and 30v in patients with non-specific CLBP at immediate and short-term follow-up compared to a simulated control group. The main discovery was that the application of 9 sessions of PRF-capacitive application was as effective at 3 weeks post-intervention as it was in a two-month follow-up for the improvement of disability, kinesiophobia, isometric abdominal muscle endurance, lumbar anteflexion mobility, quality of life, and quality of sleep. However, pain intensity only obtained statistically significant results at 3 weeks post-intervention, but not at the two-month follow-up.

Although hyperthermia has been used for years for different musculoskeletal pathologies, such as low back pain^21,22,42,43^, myofascial pain syndrome, shoulder pain^44,45^, cervical pain^46^, or patellofemoral pain^47^, with all these studies finding similar findings to ours in pain intensity and disability, to date the therapeutic effects of the capacitive mode in chronic low back pain have not been examined in depth. In a study by Notarnicola et al.^21^ in patients with chronic low back pain, the authors applied the capacitive and resistive radiofrequency mode or also called TECAR therapy, obtaining significant differences in pain and disability in all follow-up periods. In contrast to our study, in this study, pain also obtained significant results at follow-up. The differences may be due to the fact that the authors applied radiofrequency in patients who, in addition to low back pain, had documented disc disease or disc herniation. Another difference with our study is that VAS was used indistinctly for leg or low back pain, which does not allow us to compare the results of pain intensity with those of our study. In addition, other studies on the knee joint have found improvements in function compared to a simulated control group. Similarly, a recent study in low back pain found that after diathermy intervention, pain intensity and muscle stiffness were significantly lower, with the difference that this study only applied the resistive mode^42^.

Based on the available evidence, the improvement we found with PRF-capacitive pain and disability may derive from the biological effects induced by hyperthermia. Deep tissue heating may reduce pain by promoting vasodilatation and outflow of pain mediators from the affected tissue, inhibiting nociceptive transmission by activation of A-alpha and A-beta fibres or stimulating cutaneous thermoreceptors^48^. Furthermore, muscle spasm due to musculoskeletal pain is often reduced by heat and this, in turn, may contribute to improved joint function, and thus disability^27,49,50^.

Regarding the increase in the isometric resistance of the thoracolumbar musculature and the range of movement in lumbar anteflexion. Although, other studies have found similar results applying radiofrequency to other joints^27,46^, such as Alguacil- Diego et al.^46^, who found an increase in the ROM of the right rotation of the cervical spine at 8 sessions, and Albornoz-Cabello et al.^27^ who obtained an 18° increase in knee flexion in patients with patellofemoral pain syndrome. However, evidence evaluating the effect of radiofrequency diathermy on the range of motion is scarce. It is possible that the increase in range of motion may be related to an increase in tissue elasticity after radiofrequency diathermy, given that soft tissues that are heated before stretching maintain elongation after stretching, as well as requiring less force from the patient to achieve elongation^25,44,51^.

In terms of quality of life and sleep quality, a statistically significant improvement has been found in all follow-up periods. Other authors have compared radiofrequency diathermy with a simulated control group in subjects with low back pain, finding significant reductions in pain and improvements in trunk muscle activity patterns, which could be related to benefits in performing activities of daily life^42^. Unfortunately, few studies reported on the effects of diathermy on quality of life or sleep quality in patients with low back pain. Masiero et al.^50^ have linked pain reduction with an impact on quality of life, and other studies that have combined diathermy with exercise have also found improvements in quality of life. However, there is great variability in the conduct of the studies, which does not allow comparison with our results.

Regarding fear of movement, although there are no radiofrequency studies evaluating its effects on the psychological factors of the subject with CLBP, previous studies have shown a strong relationship between the severity and persistence of pain and fear of movement or activity (kinesiophobia)^52^. The results of this study could be explained by the movement avoidance model^53^, where the reduction of pain is related to an improvement in the function and mental state of the subject. There is no doubt that the psychological and biological alterations contribute to the central sensitisation and the development of chronic pain^54^. However, we cannot confirm that the levels of pain affect the results in kinesiophobia. Future studios that relate the fear of movement to disability and other psychosocial variables in patients with chronic low back pain are recommended.

There are several limitations that must be taken into account when considering our data and subsequent interpretation. Firstly, the variables analysed were only monitored in the short and medium term. Due to the chronic nature of the pathology, a long-term evaluation of these variables would have been advisable. Secondly, there is a substantial amount of controversy in determining the most effective treatment parameters, as well as the number of sessions and their duration. It is necessary and very important to compare the effects of this technique with other treatment modalities. Finally, the results have only been verified through clinical and functional evaluations, which are the means available to most physiotherapists and the best method to know the limitations of the patient with musculoskeletal pathology.

As a strength of this research, it should be noted that to our knowledge, this is the first time that PRF-capacitive has been compared with a simulated control group in subjects with CLBP; it is also the first study to measure the effects of MDR on psychological variables such as fear of movement, quality of life, and quality of sleep, factors that could contribute to the individual’s recovery process.

## Conclusions

In summary, this study found that treatment of CLBP with a 9-session dose of PRF-capacitive produced clinically significant improvements in pain, disability, and functionality (physical outcomes) in the short term, which were also significantly more pronounced than those obtained with simulated treatment. However, the long-term role of PRF-capacitive is unclear and thus further studies are required.

## Data Availability

The data of this study is available under request to the corresponding author.

## Appendix

**CONSORT 2010 checklist of information to include when reporting a randomised trial***

**Table:**

Section/Topic	Item No	Checklist item	Reported on page No
**Title and abstract**			
	1a	Identification as a randomised trial in the title	1
	1b	Structured summary of trial design, methods, results, and conclusions (for specific guidance see CONSORT for abstracts)	2
**Introduction**			
Background and objectives	2a	Scientific background and explanation of rationale	3
	2b	Specific objectives or hypotheses	4
**Methods**			
Trial design	3a	Description of trial design (such as parallel, factorial) including allocation ratio	5
	3b	Important changes to methods after trial commencement (such as eligibility criteria), with reasons	N/A
Participants	4a	Eligibility criteria for participants	5 to 6
	4b	Settings and locations where the data were collected	5
Interventions	5	The interventions for each group with sufficient details to allow replication, including how and when they were actually administered	10
Outcomes	6a	Completely defined pre-specified primary and secondary outcome measures, including how and when they were assessed	7 to 8
	6b	Any changes to trial outcomes after the trial commenced, with reasons	N/A
Sample size	7a	How sample size was determined	8 to 9
	7b	When applicable, explanation of any interim analyses and stopping guidelines	N/A
Randomisation:			
Sequence generation	8a	Method used to generate the random allocation sequence	8 to 9
	8b	Type of randomisation; details of any restriction (such as blocking and block size)	8 to 9
Allocation concealment mechanism	9	Mechanism used to implement the random allocation sequence (such as sequentially numbered containers), describing any steps taken to conceal the sequence until interventions were assigned	8 to 9
Implementation	10	Who generated the random allocation sequence, who enrolled participants, and who assigned participants to interventions	9
Blinding	11a	If done, who was blinded after assignment to interventions (for example, participants, care providers, those assessing outcomes) and how	9
	11b	If relevant, description of the similarity of interventions	N/A
Statistical methods	12a	Statistical methods used to compare groups for primary and secondary outcomes	11
	12b	Methods for additional analyses, such as subgroup analyses and adjusted analyses	11
**Results**			
Participant flow (a diagram is strongly recommended)	13a	For each group, the numbers of participants who were randomly assigned, received intended treatment, and were analysed for the primary outcome	Flowchart
	13b	For each group, losses and exclusions after randomisation, together with reasons	Flowchart
Recruitment	14a	Dates defining the periods of recruitment and follow-up	Flowchart
	14b	Why the trial ended or was stopped	N/A
Baseline data	15	A table showing baseline demographic and clinical characteristics for each group	11 (Table 1)
Numbers analysed	16	For each group, number of participants (denominator) included in each analysis and whether the analysis was by original assigned groups	12 to 16 (Tables 2 to 4)
Outcomes and estimation	17a	For each primary and secondary outcome, results for each group, and the estimated effect size and its precision (such as 95% confidence interval)	12 to 16 (Tables 2 to 4)
	17b	For binary outcomes, presentation of both absolute and relative effect sizes is recommended	N/A
Ancillary analyses	18	Results of any other analyses performed, including subgroup analyses and adjusted analyses, distinguishing pre-specified from exploratory	12 to 16
Harms	19	All important harms or unintended effects in each group (for specific guidance see CONSORT for harms)	12 to 16
**Discussion**			
Limitations	20	Trial limitations, addressing sources of potential bias, imprecision, and, if relevant, multiplicity of analyses	19
Generalisability	21	Generalisability (external validity, applicability) of the trial findings	19
Interpretation	22	Interpretation consistent with results, balancing benefits and harms, and considering other relevant evidence	16 to 19
**Other information**			
Registration	23	Registration number and name of trial registry	5
Protocol	24	Where the full trial protocol can be accessed, if available	5
Funding	25	Sources of funding and other support (such as supply of drugs), role of funders	N/A

*We strongly recommend reading this statement in conjunction with the CONSORT 2010 Explanation and Elaboration for important clarifications on all the items. If relevant, we also recommend reading CONSORT extensions for cluster randomised trials, non-inferiority and equivalence trials, non-pharmacological treatments, herbal interventions, and pragmatic trials. Additional extensions are forthcoming: for those and for up to date references relevant to this checklist, see www.consort-statement.org.
